# Effects of deep brain stimulation of the subthalamic nucleus on patients with Parkinson's disease: a machine-learning voice analysis

**DOI:** 10.3389/fneur.2023.1267360

**Published:** 2023-10-19

**Authors:** Antonio Suppa, Francesco Asci, Giovanni Costantini, Francesco Bove, Carla Piano, Francesca Pistoia, Rocco Cerroni, Livia Brusa, Valerio Cesarini, Sara Pietracupa, Nicola Modugno, Alessandro Zampogna, Patrizia Sucapane, Mariangela Pierantozzi, Tommaso Tufo, Antonio Pisani, Antonella Peppe, Alessandro Stefani, Paolo Calabresi, Anna Rita Bentivoglio, Giovanni Saggio, Lazio DBS Study Group, Maria Concetta Altavista

**Affiliations:** ^1^Department of Human Neurosciences, Sapienza University of Rome, Rome, Italy; ^2^IRCCS Neuromed Institute, Pozzilli, IS, Italy; ^3^Department of Electronic Engineering, University of Rome Tor Vergata, Rome, Italy; ^4^Neurology Unit, Fondazione Policlinico Universitario A. Gemelli IRCCS, Rome, Italy; ^5^Department of Biotechnological and Applied Clinical Sciences, University of L'Aquila, Coppito, AQ, Italy; ^6^Neurology Unit, San Salvatore Hospital, Coppito, AQ, Italy; ^7^Department of System Medicine, University of Rome Tor Vergata, Rome, Italy; ^8^Neurology Unit, S. Eugenio Hospital, Rome, Italy; ^9^Neurosurgery Unit, Policlinico A. Gemelli University Hospital Foundation IRCSS, Rome, Italy; ^10^Neurosurgery Department, Fakeeh University Hospital, Dubai, United Arab Emirates; ^11^Department of Brain and Behavioral Sciences, University of Pavia, Pavia, Italy; ^12^IRCCS Mondino Foundation, Pavia, Italy; ^13^IRCSS Fondazione Santa Lucia, Rome, Italy

**Keywords:** Parkinson's disease, voice analysis, machine-learning, deep brain stimulation, subthalamic nucleus

## Abstract

**Introduction:**

Deep brain stimulation of the subthalamic nucleus (STN-DBS) can exert relevant effects on the voice of patients with Parkinson's disease (PD). In this study, we used artificial intelligence to objectively analyze the voices of PD patients with STN-DBS.

**Materials and methods:**

In a cross-sectional study, we enrolled 108 controls and 101 patients with PD. The cohort of PD was divided into two groups: the first group included 50 patients with STN-DBS, and the second group included 51 patients receiving the best medical treatment. The voices were clinically evaluated using the Unified Parkinson's Disease Rating Scale part-III subitem for voice (UPDRS-III-v). We recorded and then analyzed voices using specific machine-learning algorithms. The likelihood ratio (LR) was also calculated as an objective measure for clinical-instrumental correlations.

**Results:**

Clinically, voice impairment was greater in STN-DBS patients than in those who received oral treatment. Using machine learning, we objectively and accurately distinguished between the voices of STN-DBS patients and those under oral treatments. We also found significant clinical-instrumental correlations since the greater the LRs, the higher the UPDRS-III-v scores.

**Discussion:**

STN-DBS deteriorates speech in patients with PD, as objectively demonstrated by machine-learning voice analysis.

## 1. Introduction

Patients with Parkinson's disease (PD) manifest variable degrees of voice abnormalities characterized by hypophonia, mono-pitch, and mono-loudness speech, and hypophonic and hypokinetic articulation. These specific voice impairments have been collectively identified as *hypokinetic dysarthria* (1, 2). PD patients may experience voice disorders from the prodromal phase of the disease, with speech deteriorating as the disease progresses (2–6). Accordingly, it is important to investigate voice changes in PD under pharmacological as well as advanced treatments such as deep brain stimulation of the subthalamic nucleus (STN-DBS).

DBS is a well-established therapeutic option for advanced-stage patients with PD (7), as demonstrated by short- and long-term follow-up studies (7–10). Besides the well-known beneficial effects of STN-DBS on the cardinal motor symptoms in PD (i.e., bradykinesia, rigidity, and tremor), the effect of this surgical procedure on specific axial functions such as voice remains elusive (7, 11–14). Following STN-DBS procedures, the estimated prevalence of speech disorders, as a post-surgical side-effect, has been reported in PD to vary between 1% after 6 months and 70% at 3 years of follow-up (12, 15–17). Hence, STN-DBS may lead to a significant worsening of parkinsonian *hypokinetic dysarthria*, resulting in a rather different voice abnormality characterized by a hypophonic voice with a strained and spastic speech mainly associated with stuttering, as suggested by previous studies in the field (18). Therefore, DBS-related voice impairments in PD patients have been identified as DBS-related dysarthria (19).

The complexity of voice as a biological phenomenon, the heterogeneity of *dysarthria* in PD, and, finally, the variable effect of STN-DBS on the voice would therefore require more advanced techniques, including artificial intelligence that allows the analysis and dynamic combination of high-dimensional datasets of voice features (20–22). Machine learning offers a potentially useful methodology to investigate voice abnormalities, especially in complex and multifactorial neurologic disorders, including PD (2, 20, 21, 23, 24).

To date, no study has assessed voice abnormalities in a large cohort of STN-DBS patients with PD compared to chronically treated L-Dopa patients with PD through objective procedures based on machine-learning analysis. Moreover, no study has correlated the clinical and instrumental assessments of voice in patients with PD by using machine-learning output measures. Filling these knowledge gaps would be relevant for the objective recognition of voice abnormalities in STN-DBS patients with PD.

In the present cross-sectional study, we examined voice performances in a large cohort of STN-DBS and chronically treated L-Dopa patients with PD using machine-learning analysis for automatic classification purposes. Therefore, we compared voice samples recorded from STN-DBS and chronically treated L-Dopa patients as well as from healthy subjects (HS), using standardized perceptual analysis as well as advanced analysis based on machine-learning procedures. We assessed the sensitivity, specificity, positive and negative predictive values, and accuracy of all diagnostic tests and calculated the area under the receiver operating characteristic (ROC) curves. Finally, by providing an objective instrumental measure of voice impairment, the likelihood ratio (LR), for each patient based on machine-learning analysis, we also assessed possible clinical-instrumental correlations.

## 2. Materials and methods

### 2.1. Subjects

This cross-sectional study enrolled 101 patients with PD (61.9 ± 7.5 years, range 41–81 years) and 108 patients with HS (60.3 ± 10.3 years, range 42–76 years). Participants were progressively recruited during regular follow-up clinical evaluations in the outpatient clinic for movement disorders at IRCCS Neuromed and University Departments and Public Hospitals on behalf of the “*Lazio DBS Study Group*.” All participants were native Italian speakers and non-smokers. None of the participants reported bilateral/unilateral hearing loss, respiratory disorders, or other non-neurologic disorders affecting the vocal cords. The participants provided written informed consent, which was approved by the institutional ethics committee of the IRCCS Neuromed Institute (NCT04846413), according to the Declaration of Helsinki.

The clinical diagnosis of PD was made according to the current standardized clinical criteria of the International Parkinson and Movement Disorder Society (25). Symptoms and signs of PD were scored using the Hoehn and Yahr (H&Y) scale and the Unified Parkinson's Disease Rating Scale Part III (UPDRS-III) (26). The clinical (i.e., perceptual) evaluation of speech abnormalities in PD was achieved by an independent rater using the specific item (item 3.1) for speech evaluation included in the UPDRS-III scale (UPDRS-III-v) during the overall motor assessment (26). In all participants, we excluded cognitive and mood impairments potentially affecting speech production through the Mini-Mental State Examination (MMSE) (27) corrected for years of education, the Beck's Depression Inventory (BDI) (28), and the Frontal Assessment Battery (FAB) (29).

The cohort of PD included patients in the mid-to-advanced phase (H&Y scores > 2) (30) and those who were chronically treated with L-Dopa. The PD cohort included two separate subgroups of patients: the first subgroup included 50 STN-DBS patients (61.6 ± 6.6 years, range 45–75 years), whereas the second subgroup included 51 patients (62.1 ± 8.3 years, range 41–81 years) chronically treated with the best medical treatment (i.e., L-Dopa). To specifically recognize the effect of STN-DBS on voice in PD, patients were enrolled and assigned to each of the two subgroups according to the inclusion criteria, attempting to statistically match the age, gender, H&Y, UPDRS, disease duration, and the L-Dopa equivalent daily doses (LEDDs) (all measures were calculated for each patient before the enrollment in the study). All patients were evaluated clinically and instrumentally 1–2 h after the administration of their chronic dopaminergic therapy (i.e., in the ON state). All implanted patients received chronic bilateral non-directional and non-interleaving STN-DBS with stable treatment and stimulation parameters for longer than 3 months. Most of the STN-DBS received bilateral monopolar stimulation (*n* = 43), the remaining being treated with bilateral bipolar stimulation (*n* = 7). Moreover, most of the patients received DBS at a frequency higher than 100 Hz (*n* = 35), whereas the remaining patients received DBS at a frequency lower than 100 Hz (*n* = 15). Stimulation parameters were set to optimize motor symptoms and fluctuations (31, 32). DBS pulse width was set at 60 μs for all STN-DBS patients. All STN-DBS patients were evaluated, clinically and instrumentally, on stimulation (i.e., when ON DBS) and on medication. Participant demographic and clinical features (including the STN-DBS parameters) are reported in Table 1.

**Table 1:** Demographic and clinical features of healthy subjects (HS), STN-DBS and L-Dopa patients.

	**Age (years)**	**Weight (kg)**	**Height (cm)**	**BMI**	**DD (years)**	**STN-DBS (years)**	**LEDDs**	**MMSE**	**BDI**	**FAB**	**H&Y**	**UPDRS-III**	**UPDRS-III-v**	**DBS freq. (Hz)**	**DBS pulse width (μs)**	**DBS intens. (mA) dx**	**DBS intens. (mA) sn**
PD STN-DBS	61.6 ± 6.6	74.8 ± 13.7	168.7 ± 9.6	26.3 ± 4.4	15.7 ± 6.2	2.9 ± 5.2	747.3 ± 386.4	28.0 ± 2.1	9.6 ± 6.9	14.5 ± 2.8	2.7 ± 0.5	20.6 ± 8.1	2.5 ± 0.5	134.5 ± 41.1	58.1 ± 9.7	2.8 ± 1.5	3.0 ± 1.3
PD L-Dopa	62.1 ± 8.3	72.5 ± 12.0	172.1 ± 8.3	24.4 ± 3.1	14.0 ± 4.5	–	758.3 ± 317.6	28.0 ± 2.7	9.5 ± 4.0	14.8 ± 2.5	2.8 ± 0.4	24.5 ± 14.7	2.0 ± 0.6	–	–	–	–
HS	60.3 ± 10.3	68.5 ± 10.6	169.0 ± 10.1	23.9 ± 3.0	–	–	–	29.0 ± 0.8	3.3 ± 1.7	17.9 ± 1.1	–	–	–	–	–	–	–

DD, disease duration; LEDDs, L-Dopa equivalent daily doses; MMSE, Mini-Mental State Evaluation; BDI, Beck Inventory Scale for Depression; FAB, Frontal Assessment Battery; H&Y, Hoehn and Yahr Scale for assessment stage of PD; HS, healthy subjects; PD, patients with Parkinson's disease; UPDRS-III, Unified Parkinson's Disease Rating Scale part III; UPDRS-III-v, Unified Parkinson's Disease Rating Scale part III, voice impairment subitem; results are expressed as average ± standard deviation.

### 2.2. Voice recordings

**Figure 1:**
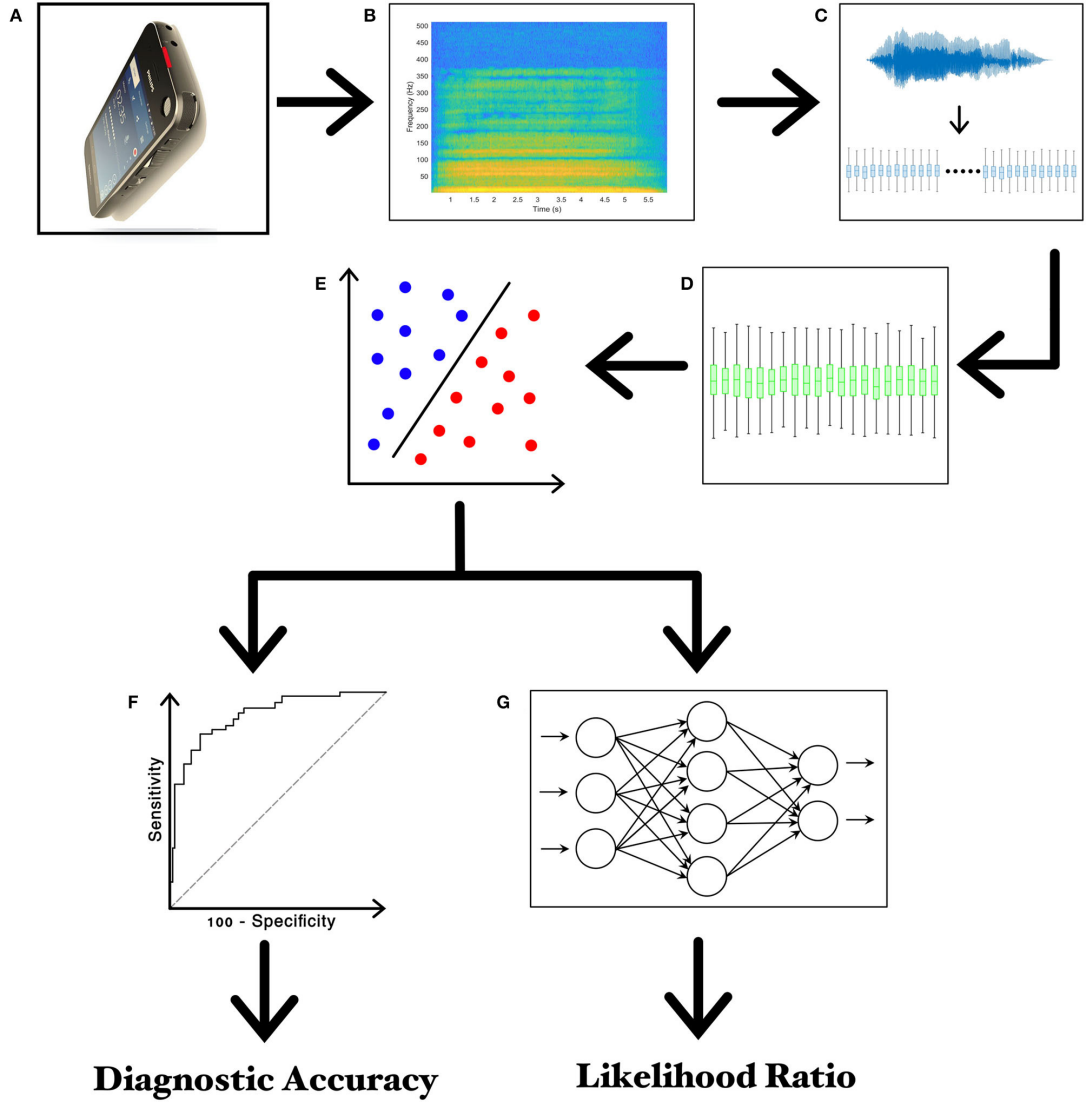
Experimental design. **(A)** Recording of voice samples through the high-definition audio recorder embedded in the smartphone; **(B)** narrow-band spectrogram of the acoustic voice signal; **(C)** feature extraction; **(D)** feature selection; **(E)** feature classification; **(F)** the receiver operating characteristic (ROC) curve analysis; **(G)** twenty-layer artificial neural network (ANN) for calculating the Likelihood Ratios (LRs).

Voice recordings were performed by asking healthy subjects and patients to produce a specific vocal task that consisted of the sustained emission of a close-mid front unrounded vowel/e/for at least 5 s (2). All audio signals were collected in a quiet and echo-free room. Voice recordings were recorded by expert neurologists. All voice samples collected in this study from controls and patients were recorded using a specific smartphone available on the market, equipped with a high-definition microphone and a dedicated application allowing for recording in linear pulse-code modulation (PCM) format (.wav) at a sampling rate of 44.1 kHz, 16-bit depth, without compressions or filtering. Participants were asked to hold the smartphone in front of their face, at ~30 cm from the mouth, and then to speak with their usual voice intensity, pitch, and quality (33) (Figure 1).

### 2.3. Machine-learning analysis

Specific spectral subtraction techniques, such as multi-band spectral subtraction, were initially used to remove background noise and other artifacts from each audio track of the voice sample. Spectral subtraction is a powerful noise reduction method based on a “learned” noise profile estimated during speech pauses and subtracted from the noisy spectrum to enhance speech. Specifically, we detected the frequency spectrum of the background noise by selecting specific sections of the audio tracks, including noise and other artifacts without biological signals (i.e., voice). The toolbox specifically employed in our analysis was the Izotope RX7 (iZotope, n.d.), which offers fine-tuning capabilities, enabling the algorithm to prioritize gating-like effects over the “musical noise” that exacerbates distortion. This procedure allowed us to reduce file corruption, possibly affecting the following analysis (34, 35).

Then, voice features underwent feature extraction and pre-processing through the Data Analytics Research and Technology in Healthcare group's Voice Analysis Toolbox (DARTH-VAT) (36). The DARTH-VAT Toolbox is open-source software provided by MATLAB (MathWorks, USA) that allows the extraction of a grand total of 345 acoustic features specific to the analysis of pathological voices. The main domains of extracted features are jitter, shimmer, HNR, glottal model-based features, empirical mode decomposition (EMD), entropy, Teager-Kaiser energy operator (TKEO), pitch period entropy (PPE), recurrence period density entropy (RPDE), and detrended fluctuation analysis (DFA) (36). Each domain entails several single-value descriptors, such as mean or standard deviation, computed as the result of a moving average on the original signal evolving in time. In addition, the DARTH Toolbox also provides additional algorithms, including SWIPE, that can extract vectors of values relative to the variation of fundamental frequency (f0) over time, such as mean, median, standard deviation, minimum, maximum, and 70% trim mean, which is the mean computed, excluding the 15% top and bottom values. Moreover, jitter, shimmer, HNR, and F0 are selected since they are common feature domains in the analysis of voice abnormalities in PD patients. Therefore, DARTH-VAT has been specifically implemented for detecting voice abnormalities in PD patients, as shown by previous research (37–39).

Moreover, extracted features underwent feature selection pre-processing using the correlation-based feature selector (CFS) (40, 41) available as an open-source toolkit in Weka (42, 43). The optimal subset was chosen with the help of a (non-greedy) Best First Search method, which involves the selection of the optimal subset and path via progressive enlargement of the cardinality while evaluating the factor of merit. The most relevant features selected by the CSF were ranked by relevance using the Information Gain Attribute Evaluation (IGAE) algorithm (44) available as an open-source toolkit in Weka (40, 41). The IGAE algorithm measures the information gained concerning the class.

After the pre-processing, the audio features underwent classification procedures. The classification focused on the 20 most relevant features, as ranked by the IGAE (23), and streamlined the data needed for machine-learning purposes (2, 20) (see Table 2). Given the relatively small dataset considered here, an SVM with a linear kernel and soft margins was used as a classifier. The SVM classifier is suitable for small datasets and noisy data since it allows for reducing the likelihood of “overfitting”. Then, we applied Platt's Sequential Minimal Optimization method to perform the supervised training of voice features (45, 46). Platt's method is an algorithm used to train SVMs and solve their quadratic programming problem. Platt's method is a fast methodology based on iteratively solving analytically small sub-problems of minimization, which only involve two Lagrange multipliers (22). The SVM was also calibrated using a logistic regressor to convert its score-like output into probabilistic values suitable for producing ROC curves. Calibration essentially works by fitting a probabilistic model to various sub-versions of the main classifier to cast the observed likelihood of their outputs into probabilities (47). However, a hyperparameter optimization was also performed to find the best-performing setup for the SVM. The main hyperparameters of the SVM are complexity (or C), which quantifies the amount of penalization for a classification error within the training set, allowing for softer or harder margins, and the ridge of the calibrator. The optimization was performed automatically owing to a look-up table of discrete values for each parameter, effectively training various versions of SVM and then posteriorly choosing the best combination of hyperparameters.

**Table 2:** Most relevant voice features selected by correlation-based feature selector (CFS) algorithm during the recording of the sustained emission of vowel/e/in healthy subjects (HS) vs. L-Dopa patients; HS vs. STN-DBS patients; and STN-DBS vs. L-Dopa patients.

**Ranking position**	**HS vs. L-Dopa**	**HS vs. STN-DBS**	**STN-DBS vs. L-Dopa**
1	DFA (Detrended Fluctuation Analysis)	F0s_median	HNR_mean
2	Jitter->F0_FM	Jitter->F0_FM	Jitter->F0_abs0th_perturb
3	mean_0th delta	Jitter->F0_TKEO_mean	Jitter->F0_TKEO_prc5
4	mean_10th delta	mean_MFCC_10th coef	Jitter->F0range_5_95_perc
5	mean_11th delta-delta	mean_MFCC_11th coef	mean_1st delta delta
6	mean_12th delta	mean_MFCC_1st coef	mean_delta delta 0th
7	mean_12th delta-delta	mean_MFCC_4th coef	mean_MFCC_4th coef
8	mean_1st delta delta	mean_MFCC_6th coef	NHR_mean
9	mean_2nd delta-delta	mean_MFCC_7th coef	Shimmer->F0_abs_dif
10	mean_4th delta-delta	PPE (Pitch Period Entropy)	Shimmer->F0_abs0th_perturb
11	mean_5th delta-delta	Shimmer->F0_abs0th_perturb	Shimmer->F0_FM
12	mean_MFCC_3rd coef	Shimmer->F0_FM	Shimmer->F0_PQ11_classical_Baken
13	mean_MFCC_6th coef	Shimmer->F0_TKEO_mean	Shimmer->F0_TKEO_mean
14	mean_MFCC_7th coef	Shimmer->F0_TKEO_prc75	Shimmer->F0_TKEO_prc5
15	NHR_mean	Shimmer->F0_TKEO_prc95	Shimmer->F0_TKEO_std
16	NHR_std	std_MFCC_10th coef	std_MFCC_10th coef
17	Shimmer->F0_FM	std_MFCC_11th coef	std_MFCC_12th coef
18	Shimmer->F0_PQ11_classical_Baken	std_MFCC_12th coef	std_MFCC_4th coef
19	Shimmer->F0_PQ5_classical_Baken	–	std_MFCC_5th coef
20	Shimmer->F0_TKEO_prc25	–	std_MFCC_6th coef

Finally, in order to improve the biological interpretation of our results by providing automatic binary discriminations among the three classes of participants (i.e., HS, STN-DBS, and L-Dopa), we identified the smallest subset of features, which were then included in further analysis. As reported in the next section of results, among the most relevant and representative extracted features, we identified Jitter.F0_TKEO_mean, Shimmer.F0_TKEO_mean, and HNR_mean. The jitter and shimmer indicate the frequency and amplitude of micro-instability in vocal fold vibrations, respectively, and both contribute to rough speech. Conversely, HNR represents the amount of noise in voice signals. In the case of our analysis, the Jitter.F0_TKEO_mean and the Shimmer.F0_TKEO_mean were both calculated as the average of the jitter and the shimmer, respectively, as computed with the aid of a Teager-Kaiser energy operator, whereas the HNR_mean was calculated as the average of the HNR.

Finally, we performed a further machine-learning analysis for clinical-instrumental correlation purposes after achieving feature extraction and selection in parallel with the SVM classification procedures. We used a feed-forward artificial neural network (ANN) consisting of a 20-neuron input layer, a 10-neuron hidden layer, and a 1-neuron output layer. Input for ANN consisted of the first 20 most relevant selected features, which thus matched the 20-neuron input layer. Then, the ANN was trained to calculate a continuous numerical value (the likelihood ratio, or LR), ranging from 0 to 1 and reflecting the degree of voice impairment in each patient with PD (i.e., the closer the LRs are to 1, the higher the degree of voice impairment). ANN was trained by using the same selected features used to train the SVM. The experimental paradigm is also summarized in Figure 1 (22).

### 2.4. Statistical analysis

The normality of all parameters was assessed using the Kolmogorov-Smirnov test. The Mann-Whitney *U*-test was used to compare demographic and anthropometric parameters in HS, STN-DBS, and L-Dopa patients. The Mann-Whitney *U*-test was also used to compare the UPDRS-III and UPDRS-III-v scores between STN-DBS and L-Dopa patients. Finally, the Mann–Whitney *U*-test was used to compare UPDRS-III, UPDRS-III-v, and LRs values in STN-DBS patients who received monopolar or bipolar stimulation as well as in patients who received low (<100 Hz) and high STN-DBS frequencies (>100 Hz).

ROC analyses were performed to identify the optimal diagnostic cutoff values to discriminate between HS vs. L-Dopa patients, HS vs. STN-DBS patients, and STN-DBS vs. L-Dopa patients. We provided detailed values for sensibility, specificity, positive predictive value (PPV), negative predictive value (NPV), accuracy, and area under the curve (AUC). Moreover, we showed the output of the ROC analysis by calculating the Youden index and its optimal criterion value, the associated criterion.

Spearman's rank correlation coefficient was used to assess correlations between clinical scores (including the STN-DBS parameters) and LR values. A *p*-value of <0.05 was considered statistically significant.

## 3. Results

Demographic and anthropometric parameters were normally distributed and comparable in HS, STN-DBS, and L-Dopa patients (all *p* > 0.05). MMSE scores were comparable among groups (all *p* > 0.05). BDI was higher, and FAB was lower in PD patients than in controls (all *p* < 0.05). Disease duration, LEDDs, MMSE, BDI, FAB, H&Y, and UPDRS-III were all similar between STN-DBS and L-Dopa patients (all *p* > 0.05) (Table 1).

### 3.1. Voice impairment in STN-DBS and L-Dopa patients

According to our results, all PD patients included in our cohort manifested a variable degree of clinically overt voice impairment (UPDRS-III subitem voice, UPDRS-III-v ≥ 1). STN-DBS patients scored higher at UPDRS-III-v than L-Dopa patients (*p* < 0.01), suggesting greater voice impairment in the first study group. When considering only STN-DBS patients, UPDRS-III and UPDRS-III-v scores were comparable in the patients who received monopolar or bipolar stimulation (*p* > 0.05). UPDRS-III was also similar in the patients who received low (<100 Hz) and high STN-DBS frequencies (>100 Hz) (*p* = 0.53). Conversely, the patients who received STN-DBS at a frequency of >100 Hz manifested higher UPDRS-III-v scores than patients treated with a frequency of <100 Hz (*p* < 0.05).

**Figure 2:**
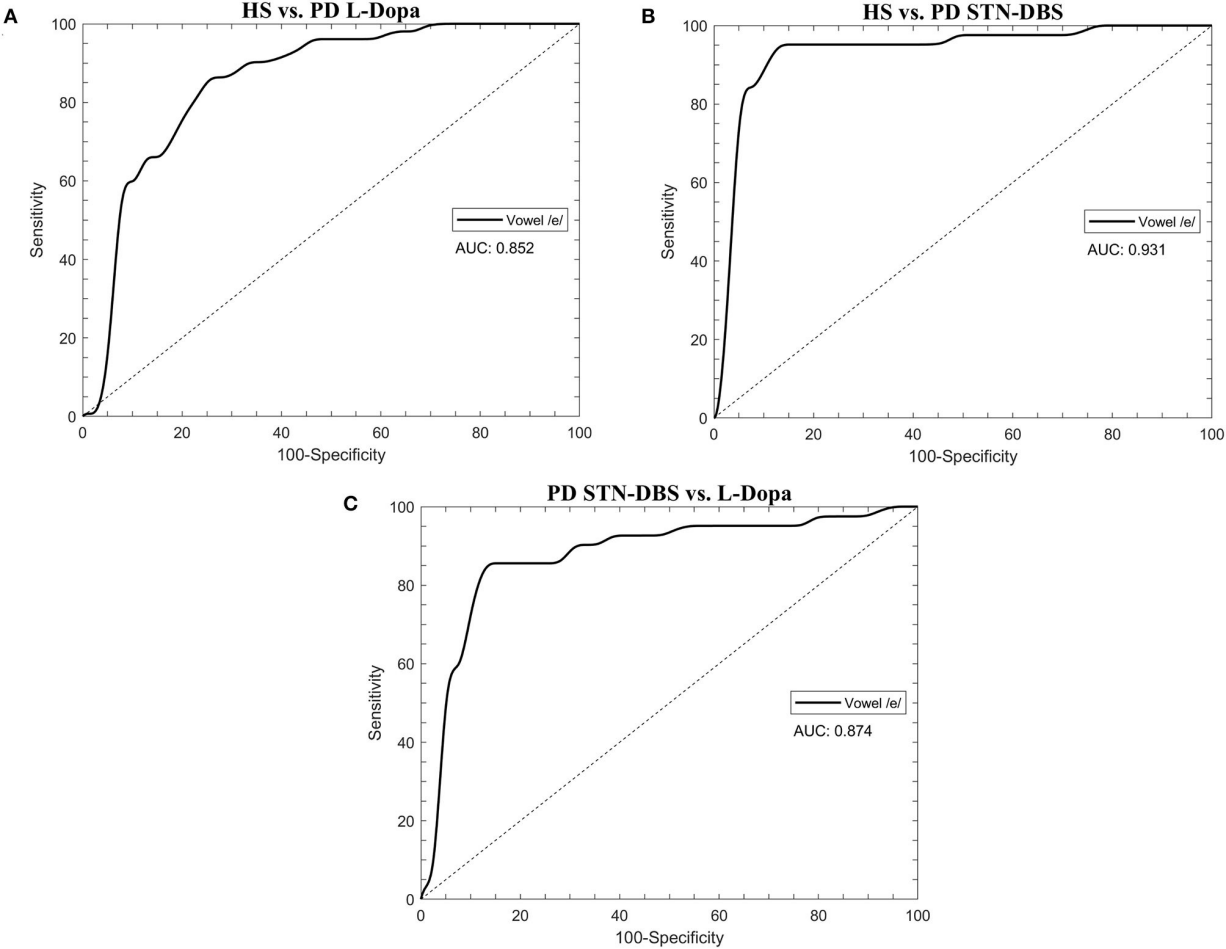
The receiver operating characteristic (ROC) curves were calculated through the support vector machine (SVM) classifier in healthy subjects (HS) and STN-DBS or L-Dopa patients with Parkinson's disease (PD). **(A)** HS vs. L-Dopa patients; **(B)** HS vs. STN-DBS patients; **(C)** STN-DBS vs. L-Dopa patients. AUC: area under the curve.

Concerning machine-learning analysis, voice samples collected from eight patients with PD (3 STN-DBS and 5 L-Dopa patients) were excluded from further analysis due to unexpected file corruption. When discriminating between HS and L-Dopa patients, the artificial classifier based on SVM allowed us to achieve a significant performance on our test. Specifically, when comparing the 20 most relevant selected features extracted from the sustained emission of the vowel, the ROC curve analyses identified an optimal diagnostic threshold value of 0.39 (associated criterion) when applying discretization and 10-fold cross-validation (Youden index = 0.63). Using this cutoff value, the performance of our diagnostic test was as follows: sensitivity = 80.0%, specificity = 78.9%, PPV = 78.4%, NPV = 80.4%, accuracy = 79.4%, and AUC = 0.852 (Figure 2A; Table 3).

**Table 3:** Performance of the machine learning algorithm.

**Comparisons**	**Instances**	**Cross validation**	**Associated criterion**	**Youden index**	**Se (%)**	**Sp (%)**	**PPV (%)**	**NPV (%)**	**Acc (%)**	**AUC**
HS vs. L-Dopa	154	10 folds	0.39	0.63	80.0	78.9	78.4	80.4	79.4	0.852
HS vs. STN-DBS	155	10 folds	0.82	0.83	88.6	95.8	95.1	90.2	92.4	0.931
STN-DBS vs. L-Dopa	93	10 folds	0.51	0.74	85.4	88.2	85.4	88.2	87.0	0.874

Performance of Support Vector Machine (SVM) linear classifier elaborating the 20 most relevant selected features during the sustained emission of the vowel/e/for three independent conditions: (1) Healthy subjects (HS) vs. L-Dopa patients; (2) HS vs. STN-DBS patients; (3) STN-DBS patients vs. L-Dopa patients. Selected features refer to the number of features able to obtain the best results; instances refer to the number of subjects considered in each comparison; cross validation refers to standardized validation procedures (see Methods for details). Se, sensitivity; Sp, specificity; PPV, positive predictive value; NPV, negative predictive value; Acc, accuracy; AUC, area under the curve.

When comparing HS and STN-DBS patients using the SVM, we achieved a significant diagnostic performance of our test, identifying an optimal diagnostic threshold value of 0.82 (associated criterion) when applying discretization and 10-fold cross-validation (Youden index = 0.83) to the 20 most relevant voice features. Using this cutoff value, we obtained the following: sensitivity = 88.6%, specificity = 95.8%, PPV = 95.1%, NPV = 90.2%, accuracy = 92.4%, and AUC = 0.874 (Figure 2B; Table 3).

When classifying STN-DBS and L-Dopa patients, the SVM applied to the 20 most relevant selected features extracted from the sustained emission of the vowel identified an optimal diagnostic threshold value of 0.51 (associated criterion) when applying discretization and 10-fold cross-validation (Youden index = 0.74). Using this cutoff value, the performance of our diagnostic test was consistent, as suggested by the following values: sensitivity = 85.4%, specificity = 88.2%, PPV = 85.4%, NPV = 88.2%, accuracy = 87.0%, and AUC = 0.874 (Figure 2C; Table 3).

**Figure 3:**
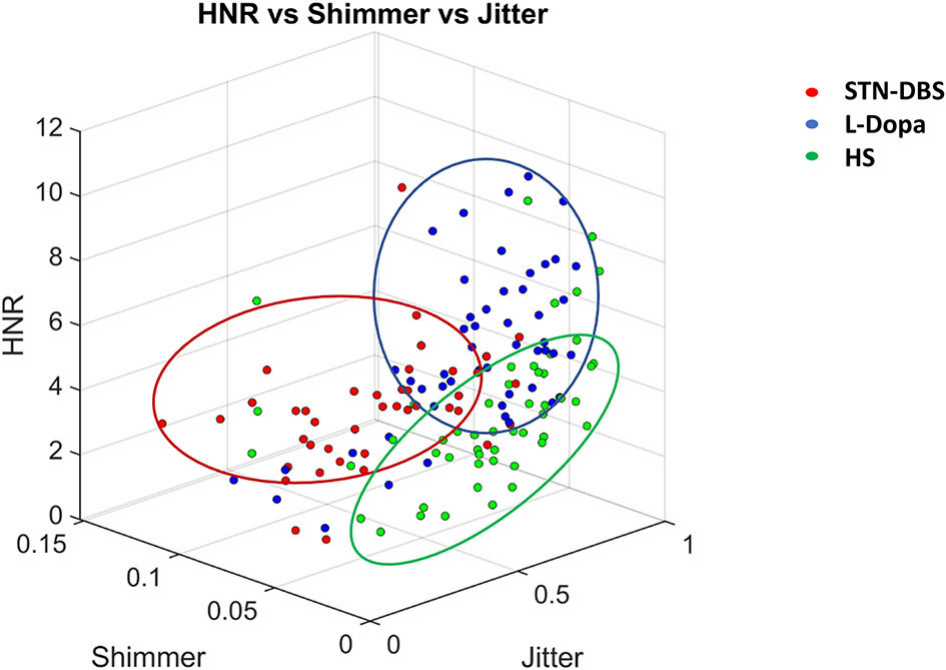
Three-D scatter plot relative to the discrimination between healthy subjects (HS), L-Dopa, and STN-DBS patients, achieved by using the three most relevant features (i.e., jitter, shimmer, harmonic to noise ratio—HNR) from those selected by machine-learning analysis. Note that the combined measurement of jitter, shimmer, and HNR allowed the discrimination of STN-DBS patients from HS and L-Dopa patients.

Concerning the analysis of binary discriminations between the three groups of classes (i.e., HS, STN-DBS, and L-Dopa), we identified Jitter.F0_TKEO_mean, Shimmer.F0_TKEO_mean, and HNR_mean, among the most relevant and representative extracted features. We achieved the following results for the L-Dopa vs. HS classification: sensitivity = 67.3%, specificity = 57.1%, PPV = 81.5%, NPV = 38.5%, and accuracy = 64.7%. Moreover, concerning the DBS vs. L-Dopa classification, we obtained the following results: sensitivity = 78%, specificity = 72.9%, PPV = 66.7%, NPV = 82.7%, and accuracy = 75%. Finally, for the DBS vs. HS classification, we reported the following significant statistic output: sensitivity = 78.3%, specificity = 75.7%, PPV = 88.9%, NPV = 58.3%, and accuracy = 77.5%. The output of this further analysis is visually displayed in a 3D scatter plot (Figure 3).

The Mann-Whitney *U*-test showed comparable LR scores in STN-DBS patients receiving bilateral monopolar or bipolar stimulation (*p* > 0.05), as well as in the patients who received low (<100 Hz) and high (>100 Hz) frequencies of STN-DBS (*p* > 0.05).

### 3.2. Correlation analysis

In L-Dopa patients, we found a positive correlation between UPDRS-III and UPDRS-III-v scores (*r* = 0.40, *p* < 0.01). A similar positive correlation between UPDRS-III and UPDRS-III-v (*r* = 0.48, *p* < 0.01) was also found when considering the cohort of STN-DBS patients. These findings demonstrate that the greater the disease severity, the higher the impairment of voice in L-Dopa patients as well as in STN-DBS. We also found that UPDRS-III and UPDRS-III-v scores did not correlate with years from the STN-DBS implant (*r* = 0.09, *p* = 0.58; *r* = 0.08, *p* = 0.54, respectively), the frequency (*r* = 0.02, *p* = 0.92; *r* = 0.14, *p* = 0.37, respectively), and the intensity of STN-DBS (mean value between the right and left STN-DBS electrodes) (*r* = 0.12, *p* = 0.45; *r* = −0.18, *p* = 0.25, respectively).

**Figure 4:**
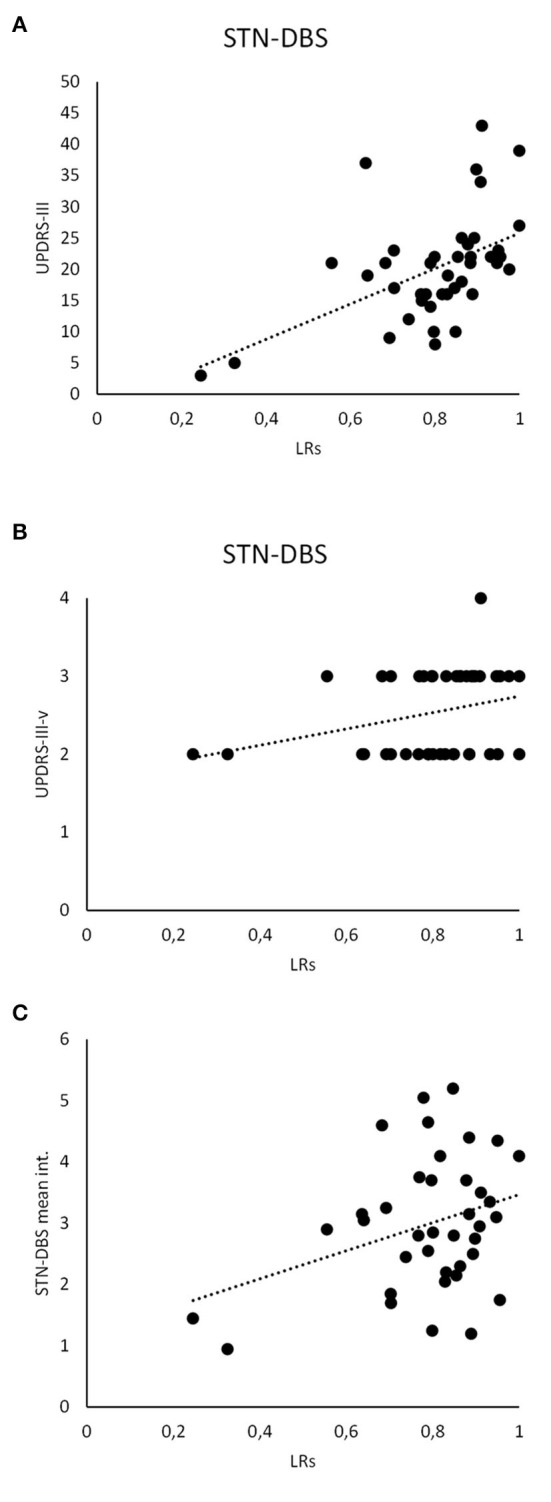
Clinical and instrumental analysis. LR scores recorded in STN-DBS patients significantly correlate with the UPDRS-III **(A)** and UPDRS-III-v **(B)**. Also, the LR scores analyzed in STN-DBS patients significantly correlate with the STN-DBS intensity (mean value between the right and left STN-DBS electrodes) **(C)**.

Concerning machine-learning analysis, we found that LR scores collected in L-Dopa patients positively correlated with UPDRS-III (*r* = 0.31, *p* < 0.05) and UPDRS-III-v (*r* = 0.41, *p* < 0.01) values. Moreover, when considering STN-DBS patients, we found a correlation between LR scores and UPDRS-III (*r* = 0.51, *p* < 0.01) as well as UPDRS-III-v values (*r* = 0.33, *p* < 0.05). Accordingly, our analysis showed that the higher the LR values calculated by machine learning, the greater the severity of motor (UPDRS-III) as well as voice (UPDRS-III-v) symptoms in both groups of PD patients (i.e., L-Dopa and STN-DBS). Finally, LR scores also correlated with the intensity (mean value between the right and left STN-DBS electrodes) (*r* = 0.33, *p* < 0.05) but not with the years from the STN-DBS implant (*r* = 0.06, *p* > 0.05) or the frequency of STN-DBS (*r* = 0.08, *p* > 0.05) (Figure 4).

## 4. Discussion

The present study provided convergent data from perceptive (i.e., clinical) as well as instrumental analysis (i.e., machine-learning), showing the effect of STN-DBS on voice in PD patients. Indeed, STN-DBS significantly worsened *dysarthria* in patients with PD, leading to DBS-related dysarthria. Supporting this conclusion, we found significant clinical-instrumental correlations between machine-learning output measures (LRs) and the clinical assessment of voice impairment (UPDRS-III-v). Our study, therefore, indicates that machine-learning analysis is a reliable tool to assess voice abnormalities objectively in STN-DBS patients with PD.

The strengths of the study include the large sample of patients and their rigorous selection based on comparable demographic, anthropometric, and clinical parameters among groups. All patients were assessed clinically and instrumentally when ON L-Dopa. STN-DBS patients were clinically and instrumentally assessed when ON DBS and ON L-Dopa, with their chronic stimulation parameters (i.e., polarity, frequency, and intensity) based on efficacy and safety on motor and non-motor symptoms, according to the best clinical practice (48, 49). The comparable LEDDs in STN-DBS and L-Dopa patients allowed us to exclude confounding factors due to dopaminergic stimulation when comparing implanted and not-implanted PD patients. The specific vocal task (i.e., sustained emission of the vowel/e/) was selected since it represents a language- and culture-free vocal task, according to previous reports (20, 21, 33, 50). All corrupted vocal samples were excluded from the analysis to avoid confounding factors due to non-biologic audio signals. Finally, our machine-learning analysis included the RASTA filtering technique, which allowed us to reduce the irrelevant and potentially misleading information added to the signal by the background noise or electromagnetic interference of the implantable pulse generator (33).

### 4.1. Clinical assessment of voice

The clinical observation that all patients manifested a certain degree of voice impairment (UPDRS-III-v ≥ 1) is consistent with the estimated prevalence of *hypokinetic dysarthria*, reaching 90% of the global PD population in the advanced stages of the disease (1, 4). Since our patients manifested higher BDI and lower FAB scores than controls, it might be argued that *hypokinetic dysarthria* also reflected a mild decline in mood and/or frontal functions. However, STN-DBS and L-Dopa patients were characterized by comparable overall disability (H&Y scores) and disease severity (UPDRS-III values), as well as BDI and FAB scores. The clinical observation that STN-DBS patients showed a higher degree of voice impairment (i.e., UPDRS-III-v) than L-Dopa patients indicates a more severe *dysarthria* in STN-DBS patients, in line with previous reports (7, 15–17, 51–54). Previous studies indeed reported prominent voice impairments characterized by a harsh, breathy, strained voice, hypernasality, imprecise consonant emission, speech rhythm disturbances, stammering, and stuttering in STN-DBS patients (13, 14, 53, 55). We also found a significant correlation between voice impairment (UPDRS-III-v scores) and overall disease severity (UPDRS-III scores), both in patients treated with STN-DBS and in those under L-Dopa, in line with previous observations (2, 13). Finally, concerning the specific STN-DBS parameters, we found that voice prominently deteriorated in patients receiving a higher (>100 Hz) rather than a lower frequency (<100 Hz) of STN-DBS. This finding t fully agrees with previous observations (13, 14, 31, 56), which outlined the well-known detrimental effect of high-frequency (>100 Hz) STN-DBS on phonatory and articulatory aspects of speech production. It is posited that high-frequency (>100 Hz) STN-DBS severely affects laryngeal coordination due to the current spreading to contiguous brain structures (57). Overall, our clinical assessment showed that STN-DBS patients manifest a significant worsening of *dysarthria* compared with those who received only L-Dopa therapy (17, 58).

### 4.2. Machine-learning analysis of voice

The accuracy achieved in discriminating L-Dopa patients from HS confirmed and expanded a recent observation from our group (2), showing that voice is altered in advanced-stage patients with PD under chronic L-Dopa treatment. This observation receives further support from the significant correlation we found between the instrumental scores (i.e., LRs) and the clinical impairment of voice (UPDRS-III-v scores) as well as motor symptoms (UPDRS-III scores) (2).

Machine learning achieved robust accuracy (92.4%) in the comparison between STN-DBS patients and controls, and the performance of the algorithm was significantly higher than that observed in the discrimination between controls and L-Dopa patients (79.4%). Moreover, machine learning achieved consistent accuracy in the comparison between STN-DBS and L-Dopa patients (87%). Again, the severity of motor (UPDRS-III) and voice (UPDRS-III-v) impairment significantly correlated with the instrumental scores (i.e., LRs) provided by the algorithm. Overall, these findings objectively demonstrate a significant worsening of voice in STN-DBS patients. Concerning the specific output of our machine-learning analysis in PD, it is worth noting that, when discriminating between STN-DBS and L-Dopa patients and, finally, healthy controls, the 20 most relevant features selected by our classifier included those reported in previous reports on spectral analysis, such as jitter, shimmer, HNR, and fundamental frequency (F0) (16, 53, 58). Further relevant biological information came from our final machine learning analysis concerning the most relevant voice features allowing discrimination among STN-DBS and L-Dopa patients and healthy controls. We demonstrated that the combination of only three independent voice features (Jitter.F0_TKEO_mean, Shimmer.F0_TKEO_mean, and the HNR_mean) allowed discrimination among the three groups of participants. Indeed, we found that jitter (i.e., the Jitter.F0_TKEO_mean) and shimmer (i.e., the Shimmer.F0_TKEO_mean) were both lower in HS than in L-Dopa and STN-DBS patients, whereas HNR (i.e., the HNR_mean) was higher in HS than in L-Dopa and STN-DBS patients. Jitter and shimmer indicate the frequency and amplitude of micro-instability in vocal fold vibrations, respectively, and both contribute to rough speech. Conversely, HNR represents the amount of noise in voice signals. Hence, we conclude that L-Dopa and STN-DBS patients are mostly characterized by abnormally rough and noisy speech compared with healthy controls. Overall, we confirm that jitter, shimmer, and HNR are very common domains in voice analysis in PD, allowing us to objectively recognize *dysarthria* in STN-DBS and L-Dopa patients.

### 4.3. Effect of STN-DBS on voice in PD: putative mechanisms

The prominent voice abnormalities observed in STN-DBS patients may reflect several mechanisms. We have recently reported that L-Dopa may improve, even though it does not restore *dysarthria* in PD (2). Following STN-DBS procedures, patients experience a significant reduction of LEDDs by ~50% (59), as a result of relevant improvements in motor and non-motor symptoms (60, 61). If not given the STN-DBS procedure, patients would have probably required at least twice the dose of L-dopa. Accordingly, following STN-DBS, our patients would be characterized by prominent voice changes simply because of decreased LEDDs. However, we did not examine voice in STN-DBS patients after a further increase of LEDDs; both implanted and non-implanted subgroups received the best medical treatment and had comparable disease stages, severity, and duration, thus making the hypothesis of suboptimal LEDDs rather unlikely. Alternatively, a mechanism for explaining the STN-DBS-related worsening of *dysarthria* in PD would imply a specific pathophysiological effect of electric stimulation on target neuronal populations. The DBS implanted in the STN may activate antidromically axons of the hyperdirect pathway (i.e., cortico-subthalamic fibers), which in turn may lead to abnormal activation of cortical areas involved in voice production, thus leading to stuttering and spastic speech (16, 19, 53, 62–64). Another reasonable mechanism would imply the spread of current from STN to contiguous brain structures owing to horizontal propagation of the electric field and the related volume of tissue activation (VTA) (65). Accordingly, STN-DBS would deteriorate voice in PD owing to the spread of the VTA to the descending corticobulbar and corticospinal tracts (53, 54, 58, 66). Moreover, an additional mechanism would imply the propagation of VTA to ascending fibers traveling in the cerebellothalamic and pallid-thalamic radiation, including those in the adjacent medial Zona Incerta, Hassler's pre-lemniscal radiation, and Forel's prerubral field or H-field (16, 17). Finally, an alternative hypothesis of DBS-related dysarthria would imply the lead location of DBS within the STN in our PD patients, as suggested by previous reports showing differential motor outcomes following the stimulation of the posterolateral/dorsomedial portion of the STN (67). Although our study lacks the neuroimaging reconstruction of electrode position and VTA for each patient, the correlation we found between the intensity used for STN-DBS and LRs values (i.e., the higher the STN-DBS intensity, the greater the voice impairment) provides support to the hypothesis of STN-DBS deleterious effect on voice in PD as a result of VTA propagation to contiguous brain structures (12, 15, 17). Accordingly, we speculate that STN-DBS deteriorates voice in PD through an abnormal engagement of specific brain structures included in the human phonological loop (68). The phonological loop is a complex cortico-subcortical network that mediates speech planning, programming, and articulation and includes regions such as the inferior frontal gyrus, supplementary motor area, primary somatosensory cortex, superior temporal gyrus, and inferior parietal lobule (69, 70). The phonological loop also includes subcortical regions, such as the striatum (i.e., the putamen) and interconnected basal ganglia nuclei (69). The cortical output of the phonological loop is the laryngeal primary motor cortex and its descending projections directed to alpha-motoneurons in the brainstem structure responsible for speech articulation, such as the nucleus ambiguous (69). In patients with PD and *hypokinetic dysarthria*, previous neuroimaging studies indeed reported abnormal activation of cortical and subcortical areas included in the phonological loop, such as the supplementary motor area, inferior lateral premotor cortex, and putamen. We, therefore, conjecture that STN-DBS may deteriorate *dysarthria* in patients with PD by degrading the activity of the phonological loop, a hypothesis that requires further investigation in future studies.

### 4.4. Limitations

When interpreting our results, several limitations should be considered. We did not record vocal samples before and after surgery or examine patients in a pharmacological OFF state or with the stimulator turned off (OFF DBS). Hence, our results do not fully explain the specific interaction of STN-DBS with dopaminergic stimulation and their combined effect on the voice in PD. This will be the topic of a future study. Moreover, the variable timing of observation after surgery (2.9 ± 5.2 years) would not affect the overall interpretation of our findings since we found no correlation between UPDRS-III-v scores as well as LRs and years from the STN-DBS implant. Our artificial intelligence could not discriminate between various components of DBS-related dysarthria (i.e., spastic and hypokinetic) in patients with PD during the analysis. Thus, this will be the topic of a future study. Furthermore, in the absence of neuroimaging data allowing the reconstruction of electrode position within the STN and the resulting VTA for each patient, our new pathophysiological interpretation based on STN-DBS interference on the human phonological loop remains rather speculative. Also, we recognize that a speech task based on the sustained emission of a vowel would be judged as not sufficient for analyzing speech production thoroughly and that short language-specific sentences based on various phonological features would provide additional results. However, as demonstrated in our previous studies, vowel emission can provide diagnostic accuracies similar to those achieved by more detailed speech tasks, including the reading of sentences (2, 71, 72). Moreover, the vowel emission gives the advantage of a language- and culture-free speech task that is useful for cohorts of advanced-stage PD patients (20, 33). Hence, we believe that sustained vowel emission represents a useful task for interpreting speech-related abnormalities in STN-DBS patients.

## 5. Conclusions

We here report the first machine learning study of voice in a homogeneous and clinically well-characterized cohort of PD patients and provide instrumental evidence of significant worsening of *dysarthria* in STN-DBS patients, thus leading to DBS-related dysarthria. Owing to an accurate methodology based on a cross-sectional design, our findings demonstrate that STN-DBS exerts a relevant impact on *dysarthria*, particularly when given at high frequency and intensity of stimulation. Our observations in PD can pave the way for new approaches based on machine-learning analysis of voice associated with current steering technology or adaptive stimulation to optimize the overall management of motor symptoms and fluctuations without worsening *dysarthria* in STN-DBS patients (65, 73, 74). Future studies based on a comparative analysis between vowel emission and short language-specific sentences would also be of help in clarifying the pathophysiologic underpinnings of DBS-related dysarthria.

## Data availability statement

The raw data supporting the conclusions of this article will be made available by the authors, without undue reservation.

## Ethics statement

The studies involving humans were approved by Institutional Ethics Committee of IRCCS Neuromed Institute (NCT04846413). The studies were conducted in accordance with the local legislation and institutional requirements. The participants provided their written informed consent to participate in this study.

## Author contributions

ASu: Conceptualization, Data curation, Investigation, Supervision, Validation, Writing—review and editing, Writing—original draft. FA: Conceptualization, Data curation, Formal analysis, Investigation, Methodology, Project administration, Writing—original draft, Writing—review and editing. GC: Data curation, Formal analysis, Methodology, Project administration, Software, Supervision, Validation, Writing—original draft. FB: Data curation, Investigation, Project administration, Writing—original draft. CP: Investigation, Methodology, Writing—original draft. FP: Investigation, Methodology, Writing—original draft. RC: Investigation, Methodology, Writing—original draft. LB: Investigation, Methodology, Writing—original draft. VC: Data curation, Formal analysis, Investigation, Methodology, Project administration, Software, Supervision, Writing—original draft. SP: Investigation, Methodology, Writing—original draft. NM: Investigation, Methodology, Writing—original draft. AZ: Investigation, Methodology, Writing—original draft. PS: Investigation, Methodology, Writing—original draft. MP: Investigation, Methodology, Writing—original draft. TT: Investigation, Methodology, Writing—original draft. APi: Writing—review and editing. APe: Investigation, Methodology, Writing—original draft. ASt: Writing—review and editing. PC: Writing—review and editing. AB: Writing—review and editing. GS: Writing—review and editing.

## Lazio DBS study group

Maria Concetta Altavista (Neurology Unit, San Filippo Neri Hospital ASL Roma 1, Roma, Italy); Alessandra Calciulli (Department of Brain and Behavioral Sciences, University of Pavia and IRCCS Mondino Foundation, Pavia, Italy); Marco Ciavarro (IRCCS Neuromed, Pozzilli, Italy); Francesca Cortese (Neurology Unit, San Filippo Neri Hospital ASL Roma 1, Roma, Italy); Antonio Daniele (Neurology Unit, Fondazione Policlinico Universitario A. Gemelli IRCCS and Department of Neuroscience, Università Cattolica del Sacro Cuore, Rome, Italy); Alessandro De Biase (Neurology Unit, Fondazione Policlinico Universitario A. Gemelli IRCCS, Rome, Italy); Manuela D'Ercole (Neurosurgery, Fondazione Policlinico Universitario A. Gemelli IRCCS, Rome, Italy); Lazzaro Di Biase (Unit of Neurology, Neurophysiology and Neurobiology, Department of Medicine, Campus Bio-Medico of Rome University, Rome, Italy); Daniela Di Giuda (Nuclear Medicine Unit, Fondazione Policlinico Universitario A. Gemelli IRCCS, Rome, Italy); Pietro Di Leo (Department of Electronic Engineering, University of Rome Tor Vergata, Rome, Italy); Danilo Genovese (Neurology Unit, Fondazione Policlinico Universitario A. Gemelli IRCCS, Rome, Italy); Isabella Imbimbo (Neurology Unit, Fondazione Policlinico Universitario A. Gemelli IRCCS, Rome, Italy); Alessandro Izzo (Neurosurgery, Fondazione Policlinico Universitario A. Gemelli IRCCS, Rome, Italy); Rosa Liperoti (Geriatrics, Fondazione Policlinico Universitario A. Gemelli IRCCS, Rome, Italy); Giuseppe Marano (Institute of Psychiatry and Psychology, Department of Geriatrics, Neuroscience and Orthopedics, Fondazione Policlinico Universitario A. Gemelli IRCCS, Università Cattolica del Sacro Cuore, Rome, Italy); Massimo Marano (Unit of Neurology, Neurophysiology and Neurobiology, Department of Medicine, Campus Bio-Medico of Rome University, Rome, Italy); Marianna Mazza (Institute of Psychiatry and Psychology, Department of Geriatrics, Neuroscience and Orthopedics, Fondazione Policlinico Universitario A. Gemelli IRCCS, Università Cattolica del Sacro Cuore, Rome, Italy); Alessandra Monge (Department of Neurology, St John the Baptist Hospital, ACISMOM, Rome, Italy); Nicola Montano (Neurosurgery, Fondazione Policlinico Universitario A. Gemelli IRCCS, Rome, Italy); Michela Orsini (Clinical Psychology, Fondazione Policlinico Universitario A. Gemelli IRCCS, Rome, Italy); Leonardo Rigon (Neurology Unit, Fondazione Policlinico Universitario A. Gemelli IRCCS, Rome, Italy); Marina Rizz (Neurology Unit, Azienda Ospedaliera Ospedali Riuniti Villa Sofia e Cervello, Palermo, Italy); Camilla Rocchi (Department of Systems Medicine, University of Rome Tor Vergata, Rome, Italy); Gennaro Saporito (Department of Biotechnological and Applied Clinical Sciences, University of L'Aquila, L'Aquila, Italy Department of Neurology, San Salvatore Hospital, L'Aquila, Italy); Laura Vacca (University and Institute for Research and Medical Care IRCCS San Raffaele, Rome, Italy); Fabio Viselli, MD (Department of Neurology, St John the Baptist Hospital, ACISMOM, Rome, Italy).
